# Assessing Glucose Uptake through the Yeast Hexose Transporter 1 (Hxt1)

**DOI:** 10.1371/journal.pone.0121985

**Published:** 2015-03-27

**Authors:** Adhiraj Roy, Angela D. Dement, Kyu Hong Cho, Jeong-Ho Kim

**Affiliations:** 1 Department of Biochemistry and Molecular Medicine, The George Washington University School of Medicine and Health Science, 2300 Eye Street, NW, Washington, D. C., 20037, United States of America; 2 Virginia Bioinformatics Institute, Virginia Polytechnic Institute and State University, 1015 Life Science Circle, Blacksburg, Virginia 24061, United States of America; 3 Department of Biology, Indiana State University, 200N 7th St, Terre Haute, Indiana 47809, United States of America; University of Minho, PORTUGAL

## Abstract

The transport of glucose across the plasma membrane is mediated by members of the glucose transporter family. In this study, we investigated glucose uptake through the yeast hexose transporter 1 (Hxt1) by measuring incorporation of 2-NBDG, a non-metabolizable, fluorescent glucose analog, into the yeast *Saccharomyces cerevisiae*. We find that 2-NBDG is not incorporated into the *hxt* null strain lacking all glucose transporter genes and that this defect is rescued by expression of wild type Hxt1, but not of Hxt1 with mutations at the putative glucose-binding residues, inferred from the alignment of yeast and human glucose transporter sequences. Similarly, the growth defect of the *hxt* null strain on glucose is fully complemented by expression of wild type Hxt1, but not of the mutant Hxt1 proteins. Thus, 2-NBDG, like glucose, is likely to be transported into the yeast cells through the glucose transport system. Hxt1 is internalized and targeted to the vacuole for degradation in response to glucose starvation. Among the mutant Hxt1 proteins, Hxt1^N370A^ and HXT1^W473A^ are resistant to such degradation. Hxt1^N370A^, in particular, is able to neither uptake 2-NBDG nor restore the growth defect of the *hxt* null strain on glucose. These results demonstrate 2-NBDG as a fluorescent probe for glucose uptake in the yeast cells and identify N370 as a critical residue for the stability and function of Hxt1.

## Introduction

Metastasized tumor cells metabolize large amounts of glucose through glycolysis and produce copious amounts of lactic acid even in the presence of oxygen [1,2]. This phenomenon, termed the Warburg effect, is a hallmark of cancer [3]. The well-established elevated glucose consumption of malignant tissue forms the basis of the clinical imaging of cancer, [^18^F] FDG-PET (positron emission tomography) [4]. The budding yeast *Saccharomyces cerevisiae*, like cancer cells, prefers to ferment rather than oxidize glucose [5,6]. Since energy generation by fermentation of glucose is inefficient, the yeast cells consume the available glucose vigorously by enhancing glucose uptake through glucose transporters [7,8].

Glucose uptake is measured using non-metabolizable glucose analogs such as 3-O-methylglucose (3-OMG) and 2-deoxyglucose (2-DG) [9–11]. After taken up by cells, 3-OMG cannot be phosphorylated by hexokinase; 2-DG is phosphorylated to 2-DG-6-phosphate (2-DG-6-P) but cannot be metabolized further. Hence, radioisotope-labeled 3-OMG and 2-DG have been widely used to measure glucose uptake. Recently, however, some inherent disadvantages associated with using radioactive glucose analogs have led to the development of improved methods for measuring glucose uptake using nonradioactive substances. 2-[*N*-(7-nitrobenz-2-oxa-1,3-diazol-4-yl)amino]-2-deoxy-D-glucose (2-NBDG) is a fluorescent derivative of 2-DG, which is converted to its phosphate form and accumulates in cells [12]. Due to its non-metabolizable and fluorescent properties, 2-NBDG has been proven useful for evaluating glucose uptake in mammalian cells [13–15].

*S*. *cerevisiae* possesses at least six members of the glucose transporter family (Hxt1, 2, 3, 4, 6 and 7) with different affinities for glucose in order to cope with environmental changes in glucose availability [16,17]. Expression of several *HXT* genes (*HXT1-4*) is repressed by the Rgt1 repressor, which recruits the general corepressor complex Ssn6–Tup1 and the *HXT* corepressor Mth1 to the *HXT* promoters in the absence of glucose [18–26]. The yeast cells employ three major glucose signaling pathways—Rgt2/Snf3, AMPK, and cAMP-PKA—that operate in a highly regulated and cooperative manner to bring about glucose-induction of *HXT* gene expression by inactivating the Rgt1 repressor [7,27,28]. The yeast glucose transporters are regulated at both transcriptional and posttranslational levels: *HXT* genes are induced by the aforementioned mechanisms; Hxt proteins undergo endocytosis and vacuolar degradation when they are not needed [29, 30].

In this study, using 2-NBDG, we investigated glucose uptake through the yeast hexose transporter 1 (Hxt1). Our study was focused on whether 2-NBDG can be used as a proxy for glucose uptake in *S*. *cerevisiae* and whether 2-NBDG is transported through the putative glucose-binding residues, inferred from human glucose transporters (Gluts). Our results show that Hxt1 transports 2-NBDG in a mechanism similar to Gluts and, furthermore, that some of the putative glucose-binding residues of Hxt1 are involved in endocytosis. Also discussed is the possible roles of these residues in the stability and function of Hxt1.

## Results

### 2-NBDG as a fluorescent probe for glucose uptake in *S*. *cerevisiae*

**Fig 1:**
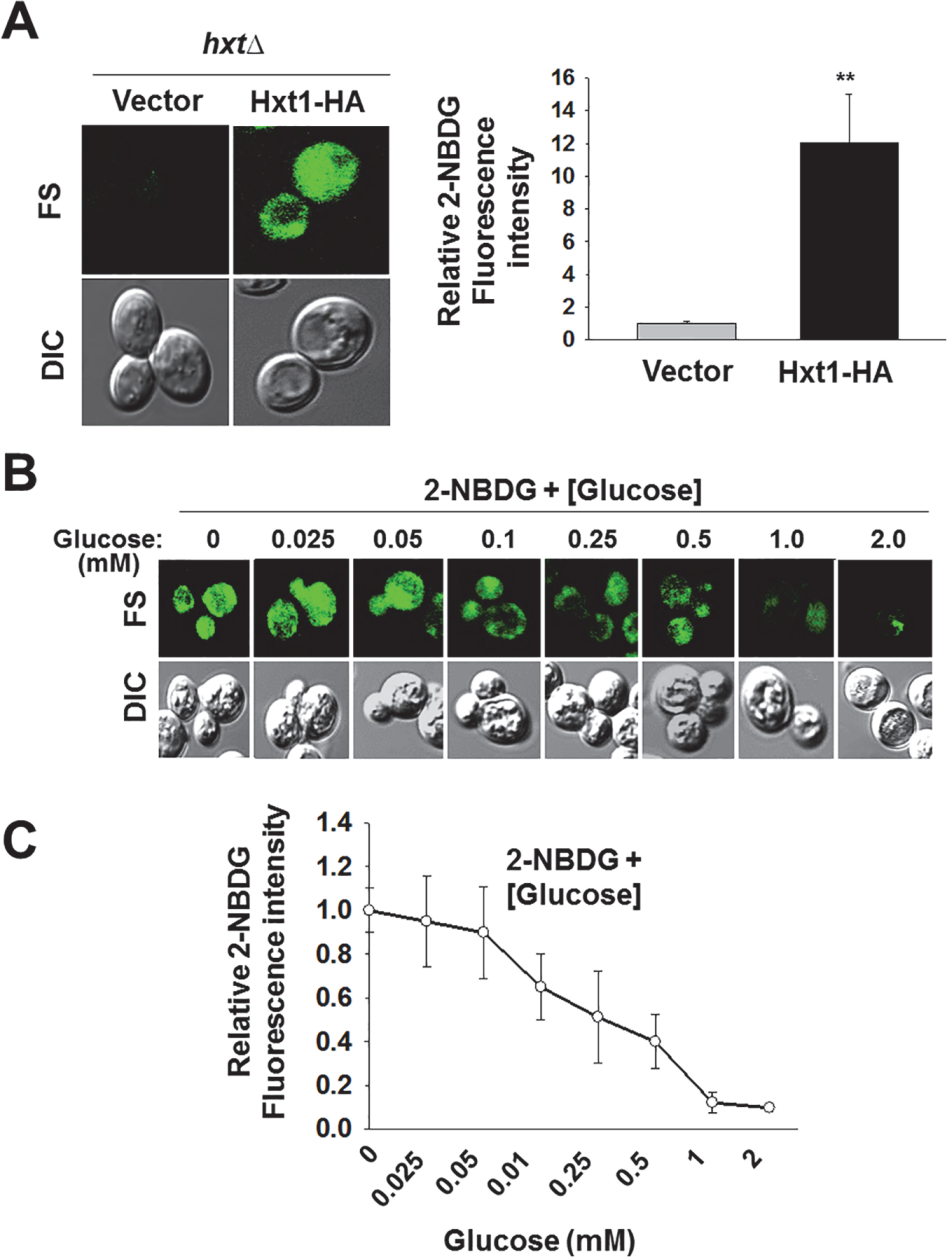
2-NBDG is incorporated into *S*. *cerevisiae* through the hexose transporter Hxt1. (A) Yeast cells (*hxtΔ*) expressing either empty vector or Hxt1-HA were first grown in SC-5% glycerol + 2% ethanol medium to mid log phase and shifted to the same medium containing 60 μM of 2-NBDG and incubated for 30 min. Fluorescence microscopy images (left panel) and quantification of relative 2-NBDG fluorescence (right panel, **P < 0.001) were shown. (B) Yeast cells (*hxtΔ*) expressing Hxt1-HA were grown as described in A. For glucose competition assay, glucose was added to different concentrations (mM) as shown and 2-NBDG uptake was analyzed by fluorescence microscopy. (C) Quantification of relative 2-NBDG uptake in glucose competition assay was shown. FL: Fluorescence, DIC: Differential Interference Contrast.

Glucose uptake assays in yeast heavily rely on the use of radioactive glucose or its derivatives, which suffer from numerous problems inherent in the use of radioactive substances. This hampers the development of facile methods for measuring glucose transport activity. 2-NBDG is often used as a fluorescent probe for glucose uptake in mammalian cells [13–15]. However, limited numbers of studies, only two early works, have shown that 2-NBDG can be incorporated into yeast cells [31,32]. To directly determine whether 2-NBDG is transported into the yeast *S*. *cerevisiae* through the glucose transport system, the yeast cells lacking all *HXT* (glucose transporter) genes [33] were transformed with an empty plasmid or with a plasmid encoding Hxt1-HA. The resulting transformants were first grown in SC-glycerol/ethanol medium till mid log phase and shifted to the same medium containing 60 μM of 2-NBDG. The intensity of the fluorescence signal was used to measure for the concentration of 2-NBDG transported into the cell. The fluorescence signal was not noticeably observed in the *hxt* null strain, but was markedly increased in the strain expressing the Hxt1 glucose transporter (Fig. 1A). Glucose competition assay was conducted to study substrate specificity by varying glucose concentrations with a fixed concentration of 2-NBDG. The results show that the fluorescence intensity is inversely correlated with increasing concentrations of glucose in the medium and that treatment of cells with 0.5 mM glucose resulted in ~ 50% decrease in the uptake of 2-NBDG (Fig. 1B and 1C). These results suggest that 2-NBDG is transported into *S*. *cerevisiae* through glucose transporters and that glucose uptake activity in yeast can be directly evaluated by measuring the incorporation of 2-NBDG into the cells.

### The yeast Hxt1 glucose transporter transports glucose in a similar mechanism as the human Glut1

**Fig 2:**
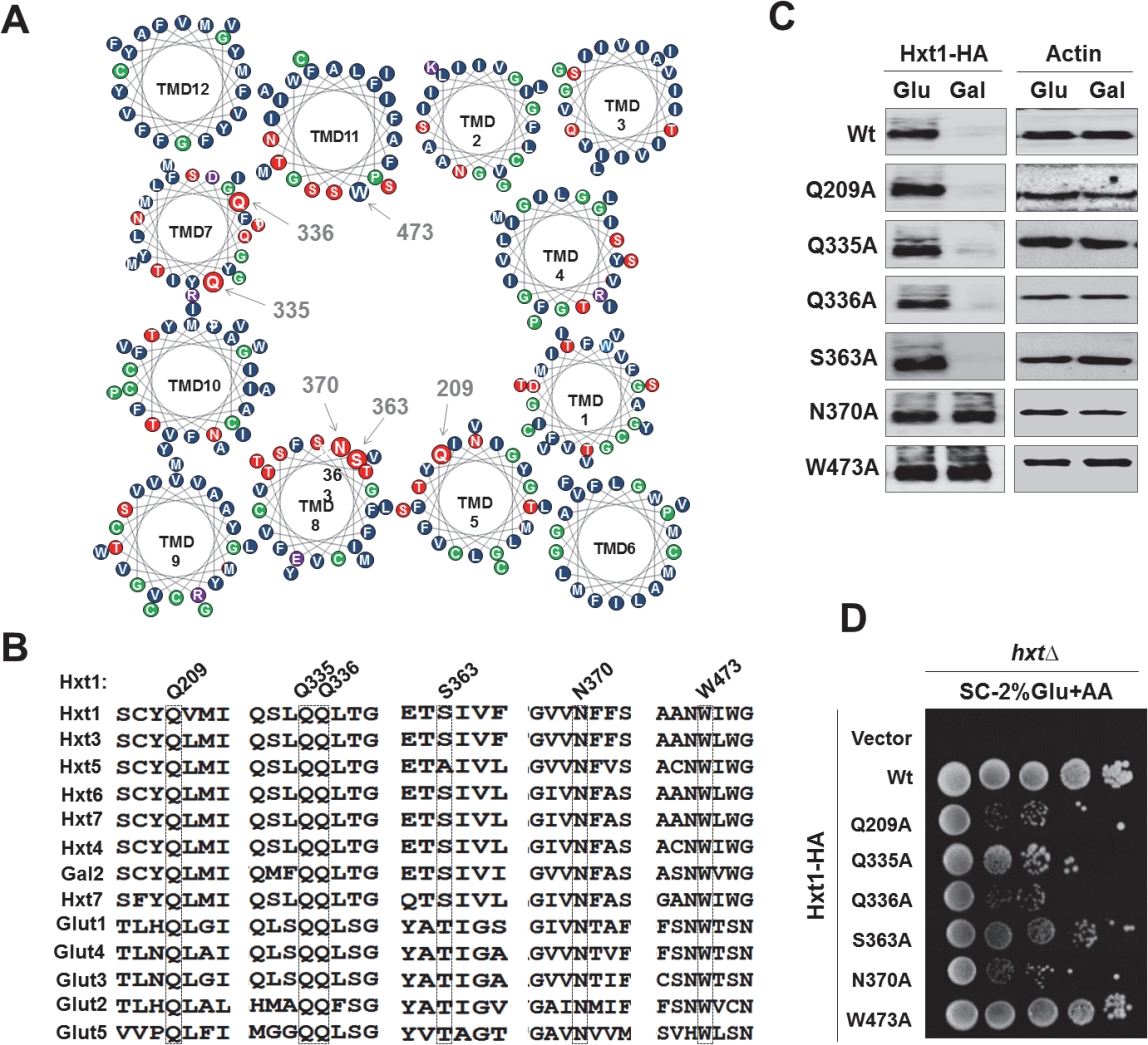
The yeast Hxt1 glucose transporter transports glucose in a similar mechanism as the human glucose transporters. (A) Cartoon for the arrangement of the 12 transmembrane helices and the proposed model of the exofacial glucose-binding sites of Hxt1 protein as viewed from the outside of the cell. For the sake of simplicity, all transmembrane helices were drawn as perfect helices perpendicular to the plane of the membrane. Amino acid residues were grouped according to their chemical properties of side chains (red: neutral polar, purple: acidic or basic polar, green: neutral nonpolar and blue: hydrophobic). Putative glucose-binding residues are shown. (B) Sequence alignment of segments of glucose transporters of yeast (Hxt1-7 and Gal2) and human (Glut1-5) showing proposed amino acids (highlighted in box) predicted to interact with glucose. (C) Western blot analysis of Hxt1-HA levels at the plasma membrane. Yeast cells (*WT*) expressing indicated Hxt1-HA proteins were grown in SC-2% glucose (Glu) medium to mid log phase and shifted to SC medium containing 2% galactose (Gal) for 6 hr. Membrane fractions were analyzed using anti-HA antibody. Actin was served as loading control. (D) Yeast cells (*hxtΔ*) expressing either empty vector or indicated Hxt1-HA proteins were spotted on 2% glucose plate supplemented with Antimycin-A (1μg/ml). The first spot of each row represents a count of 5 x 10^7^ cell/ml, which is diluted 1:10 for each spot thereafter. The plate was incubated for 3 days and photographed.

Extensive mutational analyses of human glucose transporters and crystal structures of some sugar transporters have identified residues important for glucose transport [34–41]. Since yeast and human glucose transporters are highly conserved, we examined whether yeast glucose transporters transport glucose in a similar mechanism as human glucose transporters (Fig. 2A and 2B). To this end, we first mutated the putative glucose-binding residues of Hxt1 corresponding to the residues of human Glut1—Q209, Q335, Q336, S363, N370, and W473 [42]—to alanine individually and tested the resulting mutant Hxt1 proteins for their ability to transport glucose. While the expression patterns of most of mutant Hxt1 proteins are similar to those of wild type Hxt1, the protein levels of Hxt1^N370A^ and Hxt1^W473A^ are constitutively high as compared with those of wild type Hxt1 (Fig. 2C). The *hxt* null strain is unable to grow on glucose as a sole carbon source and this defect is fully complemented by expression of wild type Hxt1 [33]. To test the mutant Hxt1 proteins for their ability to transport glucose, the *hxt* null mutant strain was transformed with plasmids encoding the mutant Hxt1-HA transporters and scored for growth on glucose medium. We observe that the growth of the *hxt* null strain is not restored by expression of most of mutant Hxt1 proteins or only partially restored by expression of Hxt1^S363A^-HA or Hxt1^W473A^-HA (Fig. 2D). Thus, these results demonstrate that the putative glucose-binding residues—in particular Q209, Q335, Q336 and N370—may be critical for glucose uptake.

### Hxt1 with mutations at the putative glucose-binding residues cannot transport 2-NBDG

**Fig 3:**
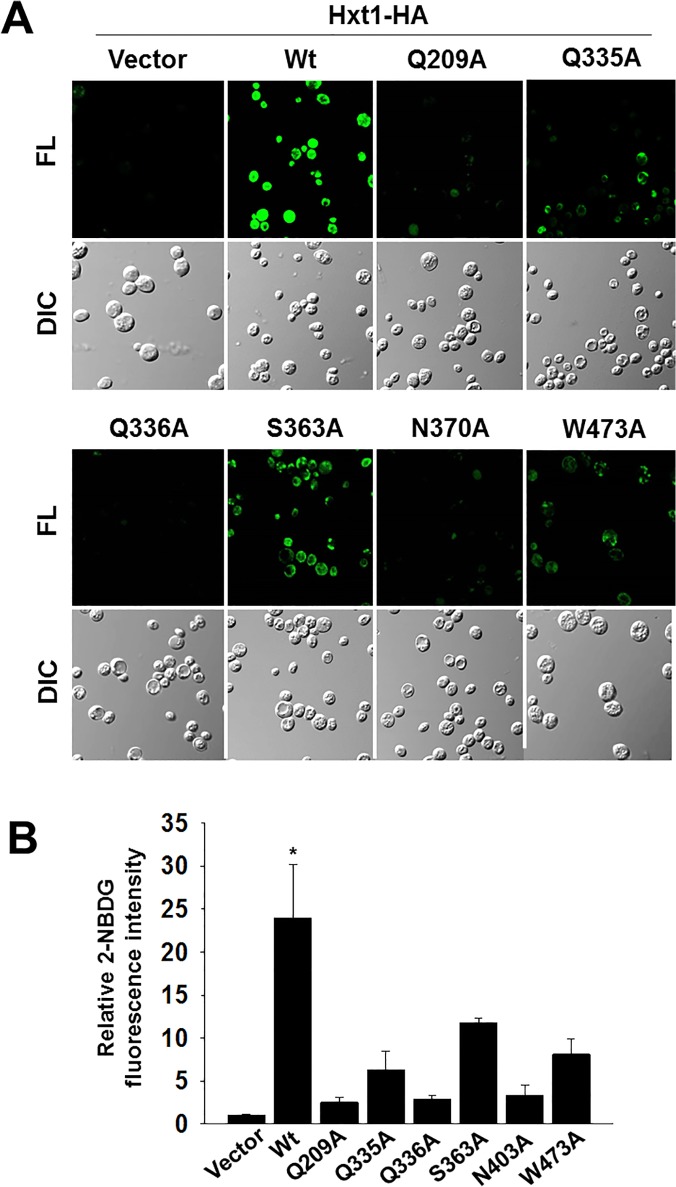
Glucose transport-defective mutant Hxt1 cannot uptake 2-NBDG. (A) Yeast cells (*hxtΔ*) expressing either empty vector or indicated Hxt1-HA proteins were grown as described in Fig. 1A. 2-NBDG uptake by the yeast cells was analyzed by fluorescence microscopy. FL: Fluorescence, DIC: Differential Interference Contrast. (B) Quantification of relative 2-NBDG uptake by the yeast cells was shown (*P < 0.05).

Given that 2-NBDG is transported into the yeast cells through glucose transporters (Fig. 1), we examined whether the putative glucose-binding residues are involved in 2NBDG uptake. To this end, the *hxt* null strain was transformed with plasmids encoding the mutant Hxt1 proteins, and the resulting transformants were treated with 2-NBDG, as described above. 2-NBDG fluorescence is hardly detectable in cells expressing Hxt1^Q209A^, Hxt1^Q336A^ and Hxt1^N370A^; by contrast, the fluorescence signals in cells expressing Hxt1^Q335A^, Hxt1^S363A^ and Hxt1^W473A^ are reduced by 50%-80%, compared with those in cells expressing wild type Hxt1 (Fig. 3A). Thus, the residues Q209, Q336 and N370 seem to be critical for 2-NBDG uptake, consistent with the above finding that these three residues may play a major role in glucose transport.

### Mutation at the putative glucose-binding residues of Hxt1 does not affect its localization to the plasma membrane

**Fig 4:**
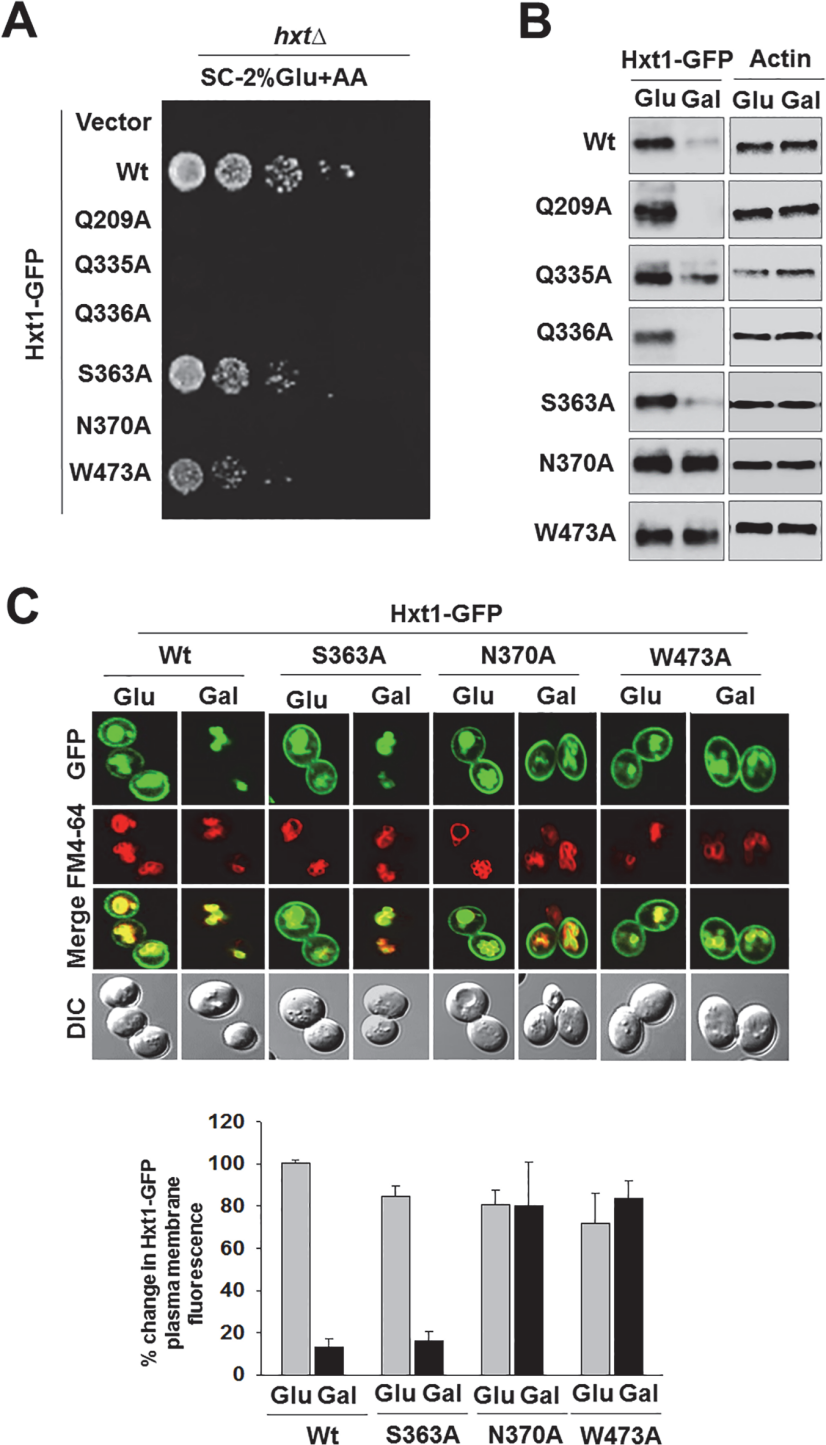
Mutation at the putative glucose-binding residues of Hxt1 does not affect its localization to the plasma membrane. (A) Yeast cells (*hxtΔ*) expressing either empty vector or indicated Hxt1-GFP proteins were spotted on 2% glucose plate supplemented with Antimycin-A (1μg/ml) as described in Fig. 2D. The plate was incubated for 3 days and photographed. (B) Western blot analysis of Hxt1-GFP levels at the plasma membrane. Yeast cells (*WT*) expressing indicated Hxt1-GFP proteins were grown as described in Fig. 2C. Membrane fractions were analyzed by Western blotting with anti-GFP antibody. Actin was served as loading control. (C) Yeast cells (*WT*) expressing indicated Hxt1-GFP proteins were grown as described in Fig. 2C and were analyzed by fluorescence microscopy (top). Relative GFP fluorescence in the plasma membrane was quantified (bottom). Relative GFP fluorescence intensities were plotted with the fluorescence of *WT* cells (2% glucose condition) set to 100%. The data represented were averages of at least 50 cell counts with error bars representing standard deviations (S.D).

Next, we determined whether mutation at the putative glucose-binding residues of Hxt1 affect its targeting to the plasma membrane by analyzing subcellular localization of GFP-fused mutant Hxt1 proteins. Expression of wild type Hxt1-GFP fully complemented the growth defect of the *hxt* null strain on glucose, suggesting that fusion of the GFP moiety to Hxt1 does not interfere with its function. However, the growth defect was not rescued by expression of the mutant Hxt1-GFP transporters or only partially rescued by expression of Hxt1^S363A^-GFP or Hxt1^W473A^-GFP (Fig. 4A). It should be noted that basal levels of glucose uptake in the *hxt* null strain expressing Hxt1-HA fusions were a little higher, compared with those in the strain expressing corresponding GFP fusions. This is presumably due to different expression levels of the two constructs; Hxt1-HA expressed from a 2μ-based, high copy number plasmid (Fig. 2) and Hxt-GFP expressed from a CEN-based, low copy plasmid (Fig. 4). Western blot analysis indicates that expression patterns of Hxt-GFP proteins are similar to those of Hxt-HA proteins: the protein levels of Hxt1^Q209A^-GFP, Hxt1^Q335A^-GFP, Hxt1^Q336A^-GFP and Hxt1^S363A^-GFP are high in glucose-grown cells but low in galactose (glucose-free)-grown cells; by contrast, those of Hxt1^N370A^-GFP and Hxt1^W473A^-GFP are constitutively high in both glucose and galactose-grown cells (Fig. 4B). In addition, fluorescence microscopy reveals that wild type Hxt1-GFP is localized to the plasma membrane in response to glucose, whereas Hxt1^N370A^-GFP and Hxt1^W473A^-GFP are found at the plasma membrane constitutively, regardless of the presence and absence of glucose (Fig. 4C). Of note, both HA and GFP fusions of Hxt1^N370A^ are expressed at high levels in both glucose-containing and glucose-free medium but unable to rescue the growth defect of the *hxt* null strain. Thus, the expression patterns of mutant Hxt1 proteins are not correlated with their ability to uptake glucose.

### Some of the putative glucose binding residues of Hxt1 are required for its endocytosis

**Fig 5:**
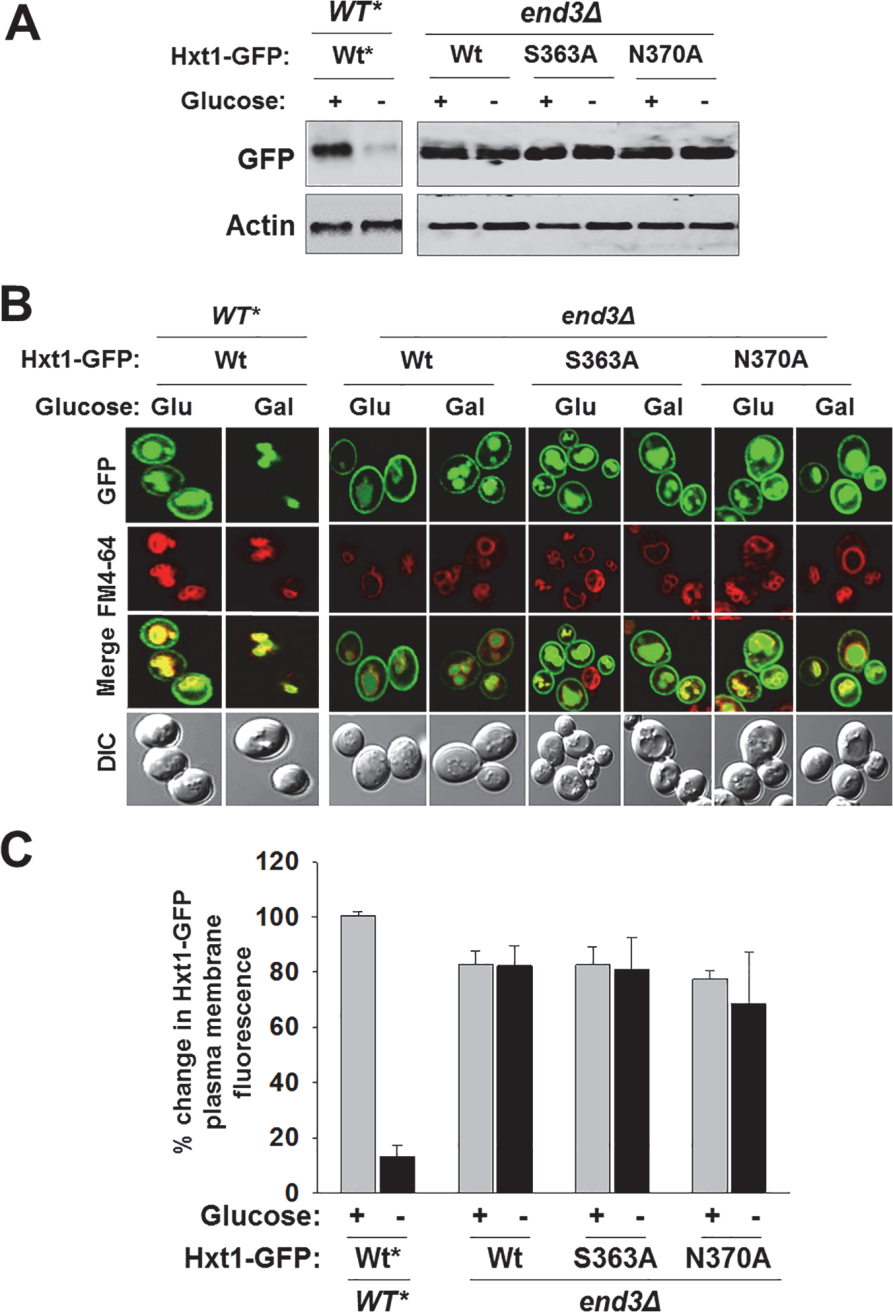
Some of the putative glucose-binding residues of Hxt1 are required for endocytosis. Yeast cells (*WT* and *end3Δ*) expressing indicated Hxt1-GFP proteins were grown as described in Fig. 2D. (A) Western blot analysis of Hxt1-GFP levels at the plasma membrane and (B) fluorescence microscopy of Hxt1-GFP proteins were shown. Actin was served as loading control. (C) Quantification of relative GFP fluorescence in the plasma membrane was performed as described in Fig. 4E. *Results of the Western blot and fluorescence microscopy analysis of wild type Hxt1-GFP protein in yeast cells (*WT*) depicted in Fig. 4C were shown for comparison.

We have recently shown that Hxt1 is endocytosed and degraded in the vacuole in response to glucose starvation [43]. Thus, it is conceivable that the reduced protein levels of Hxt1 in glucose-starved cells (e.g., galactose-grown cells) may be due to endocytosis (Figs. 2 and 4). To validate this idea, we examined endocytosis of the mutant Hxt1 proteins (Hxt1^S363A^ and Hxt1^N370A^) in a strain lacking End3, involved in the internalization step of endocytosis. We found that, in the *end3Δ* strain, the protein levels of wild type Hxt1 and mutant Hxt1^S363A^ transporters are not reduced in glucose-starved cells (Fig. 5A) and that these proteins constitutively localize to the plasma membrane (Fig. 5B and 5C). Thus, like wild type Hxt1, Hxt1^S363A^ (and presumably also Hxt1^Q209A^, Hxt1^Q335A^, and Hxt1^Q336A^) may be internalized and targeted to the vacuole for degradation in glucose-starved cells. By contrast, Hxt1^N370A^ protein levels are constitutively high in both wild type and *end3Δ* strains, suggesting that Hxt1^N370A^ (and presumably also Hxt1^W473A^) may not undergo endocytosis. Thus, these results identify N370 as a critical residue for the stability and function of Hxt1.

## Discussion

In this study, using 2-NDBG uptake assay in combination with yeast growth restoration assay, we assessed glucose uptake through the yeast hexose transporter Hxt1 and found that it may transport glucose in a mechanism similar to but subtly different from that of human Glut1 (Fig. 3B). Previous evidence suggests that Q161, Q282 and W412 of Glut1 may be involved in glucose-binding. Substitution of an asparagine at Q161significantly reduces the affinity for the substrate-binding site of a nontransported glucose analog, suggesting that this residue is critical for transport activity and exofacial ligand binding [44]. Q282 of Glut1 appears to have an important role in exofacial substrate binding but not to be critical in glucose transport, implicating the involvement of this residue in glucose-induced conformational change [45]. Our results show that Hxt1 with mutations at Q209 and Q335, corresponding to Q161 and Q282 in Glut1, respectively, is unable to restore the growth defect of the *hxt* null strain on glucose and to transport 2-NBDG into the yeast cells (Fig. 3A). Interestingly, W412 of Glut1 appears to be critical for substrate binding by interacting with the C-6 position of the pyranose ring [34,46]; however, its corresponding residue in Hxt1 (W473) is not critically required for glucose uptake (Figs. 2D and 4A). A striking feature of glucose transporters is their distinctive substrate specificity: the human Glut1 transports glucose, galactose but not fructose [47], whereas the yeast Hxt1 transports glucose and fructose, but not galactose [48, 49]. This is presumably due to differences in the sequences within and outside the glucose-binding pockets of the yeast and human glucose transporters. For example, Q282, Q283, N288, N317 and N415 of Glut1 are involved in interaction with glucose through hydrogen bonds; the first four residues are conserved in yeast transporters, whereas the aromatic asparagine at 415 in Glu1 is replaced with the non-aromatic glycine (at position 476) in yeast Hxts [41]. Therefore, it is conceivable that this difference, in part, may account for the different roles of W412 (Glut1) and W473 (Hxt1) in transporting glucose.

Yeast glucose transporters are removed from the plasma membrane and targeted to the vacuole for degradation when they are not needed. The high affinity glucose transporters Hxt2 and Hxt6/7 are endocytosed and degraded in high glucose-grown cells, whereas the low affinity glucose transporters Hxt1 and Hxt3, in glucose-starved cells [30,50–52]. We have also recently shown that the high affinity glucose sensor Snf3 is inactivated in a similar mechanism as Hxt2 and Hxt6/7 and that the low affinity glucose sensor Rgt2, as Hxt1 and Hxt3 [53]. These observations suggest that turnover of glucose transporters and sensors might be associated with their inability to bind glucose. However, the results in this study show that the mutant Hxt1 transporters unable to restore the growth defect of the *hxt* null strain on glucose—Hxt1^Q209A^, Hxt1^Q335A^, Hxt1^Q336A^ and Hxt1^N370A^—are endocytosed and degraded in response to glucose starvation except Hxt1^N370A^ (Fig. 4). Furthermore, both Hxt1^S363A^ and Hxt1^W473A^ have a reduced ability to complement the growth defect of the *Hxt* null strain, but only Hxt1^W473A^ is resistant to endocytosis (Fig. 5). Thus, it remains to be determined whether Hxt1 stability is related to its ability to bind glucose.

N370 and W473—N370 in particular—may have a pivotal role in both glucose transport and Hxt1 protein turnover. Hxt1 is ubiquitinated by the Rsp5 ubiquitin ligase prior to endocytosis [30], and often phosphorylation is a signal for ubiquitination [54–56]. We surmise that N370A and W473A mutations may induce conformational changes in Hxt1 and that the resulting mutant Hxt1 proteins are neither phosphorylated nor ubiquitinated. Consistently, a mutation of N370 of Hxt7 or N376 of Gal2, corresponding to N370 of Hxt1, to F (phenylalanine) abolishes hexose transport completely [57]. It is also noted that residues involved in the endocytosis of the Hxt1 transporter are also needed for the transport of 2-NBDG (Figs. 2 and 3), raising a possibility that 2-NBDG binds to Hxt1 and then enters the cells by endocytosis. Therefore, 2-NBDG, like glucose, is likely transported into the cells through the glucose transporter system, but the possibility of 2-NBDG uptake by endocytosis cannot be ruled out.

## Materials and Methods

### Yeast strains and Growth Conditions

The *Saccharomyces cerevisiae* strains used in this study are BY4742 (*WT*, *Mata his3Δ1 leu2Δ0 ura3Δ0 met15Δ*), EBY.S7 (*MATα hxt1-17Δgal2Δagt1Δstl1Δleu2-3*,*112 ura3-52 trp1-289 his3-Δ1 MAL2–8c SUC2 hxtΔfgy1-1* [33]) and KFY127 (*Matα his3Δ1 leu2Δ0 lys2Δ0 ura3Δ0 end3*::*KanMX* [47]). Yeast cells were grown in YP (2% bacto-peptone, 1% yeast extract) and SC (synthetic yeast nitrogen base medium containing 0.17% yeast nitrogen base and 0.5% ammonium sulfate) media supplemented with the appropriate amino acids and carbon sources.

### Plasmid Construction

The plasmids used in this study are listed in Table 1. Plasmids containing Hxt1-GFP, Hxt1 (Q209A)-GFP, Hxt1 (Q335A)-GFP, Hxt1 (Q336A)-GFP, Hxt1 (S363A)-GFP, Hxt1 (N370A)-GFP and Hxt1 (W473A)-GFP were constructed by ‘gap repair’ of BamHI-EcoRI linearized pUG35 vector. Hxt1-HA plasmid was mutagenized by QuikChange Site-Directed Mutegenesis kit (Stratagene) following manufacturer’s protocol to generate Hxt1 (Q209A)-HA, Hxt1 (Q335A)-HA, Hxt1 (Q336A)-HA, Hxt1 (S363A)-HA, Hxt1 (N370A)-HA and Hxt1 (W473A)-HA.

**Table 1:** Plasmids used in this study.

**Plasmid name**	**Description**	**Source**
JKP315	Hxt1-GFP, Ura3, CEN	This study
JKP316	Hxt1 (Q209A)-GFP, Ura3, CEN	This study
JKP317	Hxt1 (Q335A)-GFP, Ura3, CEN	This study
JKP318	Hxt1 (Q336A)-GFP, Ura3, CEN	This study
JKP319	Hxt1 (S363A)-GFP, Ura3, CEN	This study
JKP320	Hxt1 (N370A)-GFP, Ura3, CEN	This study
JKP322	Hxt1 (W473A)-GFP, Ura3, CEN	This study
pBM4527	Hxt1-HA, Ura3, 2μ	[25]
JKP326	Hxt1 (Q209A)-HA, Ura3, 2μ	This study
JKP327	Hxt1 (Q335A)-HA, Ura3, 2μ	This study
JKP328	Hxt1 (Q336A)-HA, Ura3, 2μ	This study
JKP329	Hxt1 (S363A)-HA, Ura3, 2μ	This study
JKP330	Hxt1 (N370A)-HA, Ura3, 2μ	This study
JKP331	Hxt1 (W473A)-HA, Ura3, 2μ	This study

### Yeast Membrane Preparation, Western Blotting and Protein Half-life Measurement

Membrane enriched fractions were essentially prepared as described previously [50], with some minor modifications. Briefly, after washing with phosphate buffer, pH 7.4 containing 10 mM sodium azide, the cell pellet was resuspended in ice cold membrane isolation buffer (100 mM Tris-Cl, pH 8, 150 mM NaCl, 5 mM EDTA) containing 10 mM sodium azide, protease and phosphatase inhibitors and vortexed with acid-washed glass beads. After diluting the samples with the same buffer, unbroken cells and debris were removed by centrifugation and membrane enriched fraction was collected by centrifuging the samples at 12,000 rpm for 40 min at 4° C. The pellets were resuspended in the aforementioned buffer containing 5M urea and incubated for 30 min on ice and further centrifuged at 12,000 rpm for 40 min at 4° C. The proteins were precipitated with 10% TCA, neutralized with 20 μl of 1M Tris base and finally dissolved in 80 μl of SDS buffer (50 mM Tris-HCl, pH, 6.8, 10% glycerol, 2% SDS, 5% β-mercaptoethanol). For Western blotting, proteins were resolved in 10% SDS-PAGE, transferred to PVDF membrane (Millipore) and the membranes were incubated with appropriate antibodies (anti-HA, anti-GFP or anti-Actin antibody, Santa Cruz) in TBST buffer (10 mM Tris-HCl, pH, 7.5, 150 mM NaCl, 0.1% Tween 20) and proteins were detected by the enhanced chemiluminescence (ECL) system (Pierce).

### Microscopy and Image Analysis

Yeast cells expressing Hxt1-GFP were stained with FM4-64 (lipophilic styryl dye for selectively staining vacuolar membrane, 1μg/ml) to visualize vacuole and analyzed with Olympus FluoView confocal microscope under 63X oil immersion objective lens using GFP, Texas Red filters. Images from confocal microscope were captured by FluoView software (Olympus) and un-manipulated raw images were used to quantify fluorescence intensities by ImageJ v1.4r software (NIH). For each cell in a given image, regions of interest on plasma or vacuolar membrane and in an area outside the cell (background) were traced and mean fluorescence intensities (both GFP and FM4-64) were measured. After background subtraction, the GFP signals in the plasma membranes were normalized to the FM4-64 signal of vacuolar membrane. At least 200 cells were analyzed and the data represented were the averages with error bars representing standard deviation (S.D).

### 2-NBDG Uptake Assay and Quantification

Cells were first grown in glucose free (5% glycerol + 2% ethanol) medium to mid log phase (O.D_600nm_ = 1.2–1.5). The cells were harvested, resuspended in the aforementioned medium and incubated with 60μM of 2-NBDG at 30° C for 30 min. The uptake reaction was stopped by washing the cells three times with 1X Phosphate-buffered saline, pH 7.4. Live cells were visualized with Olympus FluoView confocal microscope under 63X oil immersion objective lens using GFP filter. The quantification of 2-NBDG fluorescence was calculated using ImageJ v1.4r software (NIH). Briefly, the mean fluorescence intensity (MFI) of individual cells within the field of view was calculated and normalized by subtracting the background fluorescence signal from a region without any cells. At least 200 cells were analyzed from three independent experiments. Relative 2-NBDG fluorescence (the fluorescence of 2-NBDG from *hxtΔ* was set to 1.0) was plotted with data showing averages ± S.D.
